# Propensity to Punish in High Psychopathy may Promote Cooperation: Human and Computer Prisoner Dilemma Experiments

**DOI:** 10.1177/14747049261435215

**Published:** 2026-03-21

**Authors:** Lloyd Balbuena, Nathan Kolla, John Logan

**Affiliations:** 1 Department of Psychiatry, 7235 University of Saskatchewan, Saskatoon, SK, Canada; 2 Centre for Forensic Behavioural Science, 3783 Swinburne University of Technology, Melbourne, VIC, Australia; 3 Institute of Cognitive Science, 6339 Carleton University, Ottawa, ON, Canada

**Keywords:** game theory, evolutionary dynamics, experimental psychology, rationality, economic incentives, psychopathy

## Abstract

We tested the adaptive hypothesis of psychopathy in human and computer Prisoner Dilemma (PD) matches. From a cohort of 448 male undergraduates who completed the Levenson Self-Report Psychopathy Scale (LSRP), 31 high psychopathic (PP) and 29 low PP students, sampled from upper and lower quartiles of the cohort, played a 40-round PD match in which real money was at stake. The human matches were of three kinds: Low PP versus Low PP, Low PP versus High PP, and High PP versus High PP. In computer simulations, the empirical defection probabilities from High and Low PP human players were entered in a three-player round-robin match with tit-for-tat (TFT) or a less cooperative variant. The strategies proliferated or dwindled in subsequent generations in proportion to their winnings and the matches continued until two players became extinct. In the human matches, High PP players had higher earnings in matches with Low PP and lower earnings in matches with other High PP players. In the computer matches, TFT drove both Low PP and High PP to extinction. However, High PP drove both Low PP and a less cooperative variant of TFT to extinction. Our experiments showed that PP traits may proliferate in two ways. People with low psychopathy engage in selfish behaviors also and people with high psychopathy are capable of cooperation and deter defection by means of harsh punishment. The broader significance of the in silico experiment is that randomness in social encounters may allow behavioral expressions of psychopathy to thrive over an altruistic and reciprocal strategy such as TFT.

## Introduction

### The Adaptive Hypothesis of Psychopathy

Psychopathy, from the view of evolutionary psychology, is an adaptive life strategy for individuals when their frequency in the population is low (Ellis, 1988; Harpending & Sobus, 1987; Mealey, 2006). This is usually understood as a minority of individuals (psychopaths) in a population of cooperators (Harpending & Sobus, 1987). Here, we provide evidence for the alternative interpretation: a minority of psychopathic (PP) actions in a general ethos of cooperation. Evolutionary arguments have used the terms antisocial personality, sociopathy, and psychopathy interchangeably, resulting in a muddled discourse (Pemment, 2013). Psychopathy and antisocial personality share common genes that predispose to externalizing behaviors (Larsson et al., 2007). Sociopaths also engage in criminal behavior, but this is attributed to environmental causes such as poor parenting (Walsh & Wu, 2008). We do not distinguish between these terms because the distinctions probably reflect the differentiation of academic disciplines instead of the phenomenon itself. Deficient emotions (psychopathy), criminality (antisocial personality), and moral deviance (sociopathy) may each confer an evolutionary advantage in a particular niche.

PP traits can be advantageous for sexual reproduction. PP individuals misrepresent themselves to women as sincere and committed (Tooke & Camire, 1991). They have low fluctuating asymmetry (Lalumière et al., 2001), and a “precocious sexuality” geared towards reproductive success (Harris et al., 2007). Physical symmetry is desirable in potential mates and deviations from it may reflect birth defects or environmental insults (Thornhill & Moller, 1997). Lower FA was associated with short-term, uncommitted relationships, and greater willingness to engage in dominance, and self-promotion (Simpson et al., 1999), which are traits associated with psychopathy. Low fear (vs. shyness), charm, and glibness are advantages in sexual reproduction (Harpending & Sobus, 1987; Mealey, 2006). Impersonal sex and the lack of remorse can insulate a psychopath from distress and heartbreak. Romantic failure can trigger suicidal behavior (Kazan et al., 2016) yet suicide is rarely carried out in psychopaths (Cleckley, 1976).

Although the term psychopath conjures the image of a violent criminal, most PP people are not violent. Taxometric studies indicate that psychopathy is best understood as a dimensional construct rather than a discrete category (Haslam et al., 2020). Many individuals who display elevated levels of callousness, shallow affect, or manipulativeness may function adaptively within society without engaging in violence. PP people held roles such as single contributor, support staff, and management in public and private organizations (Eisenbarth et al., 2018). Using the Psychopathic Personality Inventory-Revised, Eisenbarth et al. (2018) found that self-centered impulsivity and fearless dominance were negatively and positively related to professional satisfaction, respectively. Being a successful psychopath may require the ability to regulate aggression and antisocial tendencies as a compensatory mechanism (Lasko & Chester, 2021). The term “compensatory” indicates a mitigation mechanism that does not remove the defect. As Cleckley observed, “the psychopath has a very serious disability, disorder, defect, or deviation (Cleckley, 1976, p. 367).”

### The Prisoner Dilemma as an Experimental Model of Adaptiveness

The social dynamics of psychopathy are difficult to study directly but the Prisoner Dilemma (PD) is exceptionally well-suited. For readers unfamiliar with the PD, the following scenario motivates the nomenclature (Poundstone, 1992).

Bob and Gord robbed a bank and are at the police station where they are interrogated in separate rooms. The police have insufficient evidence to charge them, so each one is being offered to turn against the other by confessing in exchange for leniency. If Bob confesses and Gord does not, Gord gets prison time while Bob gets a lighter sentence. If the reverse happened and Gord confessed but Bob did not, Bob gets prison time while Gord gets the lighter sentence. If both kept silent, both are released because of insufficient evidence. If both confess, they both get prison time.

Theory suggests that PP people would be more likely to confess (“defect” in game theory parlance) because of greater selfishness, a belief in their ability to manipulate the police to extract favorable terms, and shallow affect gives them no compunction in betraying their associate. In the company of other bank robbers, PP people may deny the betrayal. Since it would be ethically dubious to conduct a real-life experiment with robbery suspects and police, the PD is played as a laboratory experiment in which incentives and disincentives substitute for prison time and leniency. These incentives take the form of varying amounts of money that in turn depend on the combination of choices made by the players (see Table 1).

**Table 1.** Payoff Structure in Our Seesaw Payoff Variation of the Prisoner Dilemma.

	Cooperate	Defect
Cooperate	Reward (2.5, 2.5)	Sucker (0, 5)
Defect	Temptation (5, 0)	Punishment (1, 1)

Over the years, numerous studies PD experiments have been conducted to study PP behaviors. Mokros and colleagues (2008) had 24 male PP inpatients (including 10 inmates) and 24 males from the general population play against a computerized opponent that used a tit-for-two-tats strategy. Tit-for-tat (TFT) can be defined as: cooperate first and copy the opponent's previous move thereafter (Rapoport et al., 2015). The PP players exhibited more selfish behavior by defecting more often than general population counterparts. (See the payoff matrix under Methods for a definition of “defect.”) Similarly, Balafoutas and colleagues (2021) recruited prison inmates to participate in games played for money. These games were the Trust Game, the Equality Equivalence Test, the Corruption Game, and the PD. Inmates with higher psychopathy were less likely to engage in reciprocity, less likely to cooperate in the PD, and more willing to accept bribes in exchange for favors.

Testori and colleagues (2019) examined the effect of group composition on the rates of cooperation in the PD by experimentally manipulating the mix of high- and low-PP people. They found that a higher ratio of high-low PP players decreases average levels of cooperation in the group. This result supports the claim that psychopathy is adaptive for the individual at low frequencies (Dugatkin & Wilson, 1991; Mealey, 2006) because a greater number of cooperators represents more opportunities for psychopaths to exploit. In the repeated PD (i.e., having more than one round), low cooperation results in lower earnings compared to high cooperation, which suggests that games played between two high PP people result in lower average earnings for the pair compared to games played between low PP people (Colman & Wilson, 1997; Harpending & Sobus, 1987). The present study compared these two conditions.

Baggio and Benning (2022) demonstrated that fearless dominance (the lack of fear in interpersonal encounters) in psychopathy is advantageous when the opponent is forgiving (i.e., cooperates after the psychopath's defection) but disadvantageous when the opponent is harsh. In effect, PP people may calibrate their selfishness to fit the opponent's propensity to inflict punishment. It has also been shown that PP individuals have a capacity for cooperation (Widom, 1976), perhaps with extra effort (Rilling et al., 2007) and conditional on their appraisal of the opponent's value in future interactions (Gervais et al., 2013). Taken together, these results suggest that PP people pursue a selfish strategy in the PD but adjust their playing style when opponents retaliate.

The emotional responses of players in the PD have been studied extensively in a series of experiments by Rilling and colleagues. First, they found that mutual cooperation is associated with higher activity reward-related brain areas (Rilling et al., 2002). Subsequently, they found that when a player cooperates and this is unreciprocated by the opponent, the anterior insula is activated, reflecting distress and anger (Rilling et al., 2008). Interestingly, they also found that compared with low PP players, high PP ones had a weaker aversion to unreciprocated cooperation (Rilling et al., 2007). Defection by high PP individuals was associated with less guilt as shown by lower activation in the dorsolateral prefrontal cortex and rostral anterior cingulate cortex. These results indicate that humans have psychological mechanisms that promote reciprocity and disincentivize selfishness. However, these mechanisms may be weaker in PP individuals.

### Computer-Based PD Matches

Computer-based PD matches are a valuable complementary body of research because human PD matches cannot model population dynamics, by which is meant how the frequency of PP players changes over time as a function of their winnings. The downside is that the strategies in computer PD matches do not reflect how humans actually play. In general, the strategies in game theory aim to maximize winnings without considering human cognitive limitations (Selten, 1990). However, unlike human matches, computer matches can show how one strategy becomes fixed in the population. For example, in a single round PD model, defection cannot be beaten (Axelrod & Hamilton, 1981) and cannot be driven out in the population. But in the repeated PD, TFT consistently beats other strategies (Axelrod & Hamilton, 1981), and it has been suggested that TFT is as a catalyst for cooperation (Nowak & Sigmund, 1992). Its success is attributed to niceness (by not defecting first), reciprocating cooperation and defection consistently, and predictability to the opponent (Rapoport et al., 2015).

### What This Work Adds to the Literature

The gap in the literature this paper seeks to fill is the bifurcation of two strands of research about selfish behavior in psychopathy. Lacking empathy, psychopaths have limited restraint over their natural inclination for selfish behaviors (Blair, 2007; Hare, 1993). In a sense, selfishness represents a fork in the road between psychology and economics because the latter considers selfishness the basis of rationality. As Adam Smith (2015) eloquently observed, “It is not from the benevolence of the butcher, the brewer, or the baker that we expect our dinner, but from their regard to their own interest.” Tying these two research strands has implications for the debate about whether psychopathy can be cured or eradicated. If psychopathy is a clinical disorder, does it nonetheless represent a viable economic strategy that explains its persistence?

In this work, we designed human and computer experiments to test these contrasting perspectives. We first had human PD matches and then simulated the strategies of high and low PP players (henceforth High PP and Low PP) in computer matches. Our study requires two assumptions: (1) monetary earnings in the human experiment are a proxy variable for real-life success, (2) For the computer matches, higher earnings in one generation causes proliferation (social or biological) in the subsequent one (Maynard Smith, 1982). We hypothesized that: (1) Low PP players would defect less frequently (cooperate more often) than High PP players, (2) High PP players paired with Low PP would win more money compared to High PP paired with fellow High PP players. We did not have a hypothesis for our computer simulation.

## Methods

### Payoff Matrix

As can be seen from Table 1, the players have one of two choices, “Cooperate” or “Defect.” Henceforth, we use the terms “cooperating” of “defecting” to indicate the choice made by the player. The choices need not be labeled “Cooperate” or “Defect” as such for it is the payoff structure that defines the PD and not the labels. In the classic PD, the payoffs for the row player obey the following four constraints (Axelrod & Hamilton, 1981):

1. Temptation > Reward
2. Reward > Punishment
3. Punishment > Sucker
4. 2 × Reward > Temptation + Sucker

The payoffs (Table 1) satisfy the first three conditions qualifying it as a PD (Hirshleifer & Coll, 1988; Kuhn, 2019). We changed the fourth condition such that 2 × Reward = Temptation + Sucker. We call this change the *seesaw* payoff variation. The intuition behind it is: “If I exploited you by mistake in one round, I could undo the damage by allowing you to exploit me in the next, if so desired.” Thus, it allows for the Pareto optimal outcome over 40 rounds to be also achieved by alternating exploiter and exploited roles, as suggested by Rapoport and Chammah (1965). It is also related to Nowak and Sigmund's (1994) alternating PD strategy that allows for the correction of mistakes and restores cooperation. Table 1 payoffs applied both to human and computer experiments.

## Human Experiment

*Participants.* The recruitment of participants involved two stages: administering a psychopathy instrument to 1,412 Introductory Psychology students at an eastern Canadian university. Ethics approval was granted by the university ethics board. The students took part in an online mass testing activity for which they received academic credit. The battery of tests included the Levenson Self-Report Psychopathy scale (LSRP) (Levenson et al., 1995). The LSRP consists of 26 statements that the test-taker rates on a four-point scale with the following values: 1 (Strongly disagree), 2 (Disagree), 3 (Agree), 4 (Strongly agree). Seventeen of its items are positively worded: “Making a lot of money is my most important goal”; nine items are negatively worded: “I would be upset if my success came at someone else's expense.” The latter were reverse coded after which all items were summed. The total score has a range of 26–104.

The LSRP was chosen because it is appropriate for non-institutionalized samples and has been factor analyzed to replicate the two-factor structure of the Psychopathy Checklist-Revised (Kosson et al., 2013; Salekin et al., 2014), the gold standard measure of psychopathy in forensic settings (Brinkley et al., 2001). Briefly, primary psychopathy is related to selfishness and interpersonal manipulation while secondary psychopathy is related to impulsivity and antisocial actions (Levenson et al., 1995). None of the 26 questions directly asks the respondent if they committed an illegal or antisocial action. Instead, the person's world view is elicited, as shown in the item, “I often admire a really clever scam.” The LSRP has been validated in non-North American samples or non-English speakers (Ghossoub et al., 2024; Maheux-Caron et al., 2020; Shou et al., 2017). Previous studies also used the LSRP in prison inmates (Balafoutas et al., 2021) and in university students (Johnston et al., 2014). Other studies have used the Psychopathic Personality Inventory-Revised in prison inmates (Mokros et al., 2008) and in a non-incarcerated sample (Testori et al., 2019). Regardless of the instrument used, high psychopathy was related to lower cooperation in these studies. For this study, we were interested in the total LSRP scores only, but the primary and secondary scores are also reported in Table 2.

**Table 2.** Participant Characteristics.

Mean (SD)	High PP (n = 31)	Low PP (n = 29)	t-Test of the Difference (*p*-Value)
Age	19.00 (1.21)	21.14 (5.47)	−2.12 (.04)*
LSRP Total	65.68 (6.35)	45.03 (2.77)	16.13 (<.001)
LSRP Factor 1	39.97 (4.20)	26.15 (3.12)	14.04 (<.001)
LSRP Factor 2	26.07 (3.99)	18.52 (3.09)	7.94 (<.001)
Tower of Hanoi (moves)	30.63 (17.33)	32.86 (18.95)	−0.47 (.64)
Tower of Hanoi (time in secs.)	72.83 (55.79)	85.10 (66.08)	−0.77 (.44)
Total points won	73.48 (19.69)	74.00 (21.75)	−0.10 (.92)

Of 448 male test-takers, 117 scored in the high quartile and 110 in the low quartile, High PP and Low PP, respectively. The internal reliabilities in male test-takers were 0.86 for both the total score and for Factor 1, and 0.63 for Factor 2. We then sampled 60 students, from upper and lower quartiles, to maximize the difference in trait levels. This two-stage sampling design results in greater power to detect behavioral differences compared to a simple random sample of similar size (Cochran, 1977). Specifically, we calculated that to obtain a difference in mean LSRP scores equal to what is reported in Table 2 (about 20.65 points), 168 people would have been required (84 per group) under random sampling based on formulas for the variance of the difference in means (Cochran, 1977) and relative efficiency (Kish, 1965).

The sample was restricted to males to avoid confounding by sex, as some research suggests that females with High PP traits are less sensitive to unfairness than males (Efferson & Glenn, 2018). Widom (1976) also previously used an all-male sample to study psychopathy in the context of a PD. The students were invited to sign up for an experiment advertised as *Personality Traits in Games Played for Money*. A total of 60 students participated in the PD with our modified payoffs (Table 1). The Tower of Hanoi task was also administered to examine differences in cognitive ability. We were simply interested in participants’ ability to understand how the amount to be won depends on the joint actions of oneself and the other player, and not fine-grained differences in IQ or executive function. The Tower of Hanoi task was administered to adjust for potential differences in executive function. In this task, there are four or five towers and the objective is to move rings in the left-most tower to the right-most tower subject to the constraint that only one ring can be picked up at a time and a ring cannot be put on a larger ring. As shown in Table 2, there was no difference by time or number of moves between Low PP and High PP groups. Please refer to Appendix I for the Tower of Hanoi screen layout and instructions.

*Administration.* Three undergraduate psychology students who were blind to the PP and demographic backgrounds of the participants were trained as research assistants and they administered the experiments. Participants were later debriefed about the purpose of the experiment (i.e., to relate winnings with PP traits) and they were given a chance to have their data purged. None chose to withdraw consent. The profiles of student participants are described in Table 2. The two groups were well-matched except that Low PP students were about 2 years older.

*Procedure:* Two students at a time played against each other using the *Econport* platform (Chen et al., 2003) accessed through a laptop. These students were seated with backs to each other in a quiet room with an administrator present. They were not allowed to communicate during the game. Each student was told to select one of two choices labeled [S, C], while the other participant did so independently. After each round, a message was displayed on screen that showed accumulated winnings: Your balance: xx. There were 40 rounds with the same partner. There were 8 Low PP–Low PP pairs, 9 High PP–High PP pairs, and 13 mixed pairs.

Participants were compensated with a base amount of $5 plus 1 cent for every point won in the PD, in addition to 2 percentage points of academic credit for participation. The base amount and academic credit were unconditional on game performance. Appendix II contains the instructions to the participants and hands-on practice that tested for understanding.

*Variables of interest.* The two dependent variables corresponding to our hypotheses were: defection odds and points won. Defection odds is the ratio of selfish to cooperative choices. The explanatory variables were player type (High PP or Low PP), opponent type (High PP or Low PP), whether the opponent defected in the previous round, and round (or trial). This last variable reflected whether players were more likely to defect (or earn more points) as the match progressed. In the points won model, pair type was entered as an independent variable. This was a dichotomous variable with levels 0 = at least one player was Low PP, and 1 = both players High PP. This coding was based on Testori et al.'s finding that the level of cooperation varied inversely with the number of High PP players. We were interested in whether High PP players paired with fellow High PP players earned less.

*Statistical models.* For defection, a logistic regression model with random effects and robust standard errors was created, while a linear regression model was created for points won. Robust standard errors adjust for correlated choices within a participant. In both models, a random effect for participant was entered. These models were implemented using the Stata commands xtlogit and xtreg. The Stata code used in the analysis is available at https://github.com/lloydxeno/PrisonerDilemma/blob/main/human.

## Results of the Human Matches

**Figure 1.**
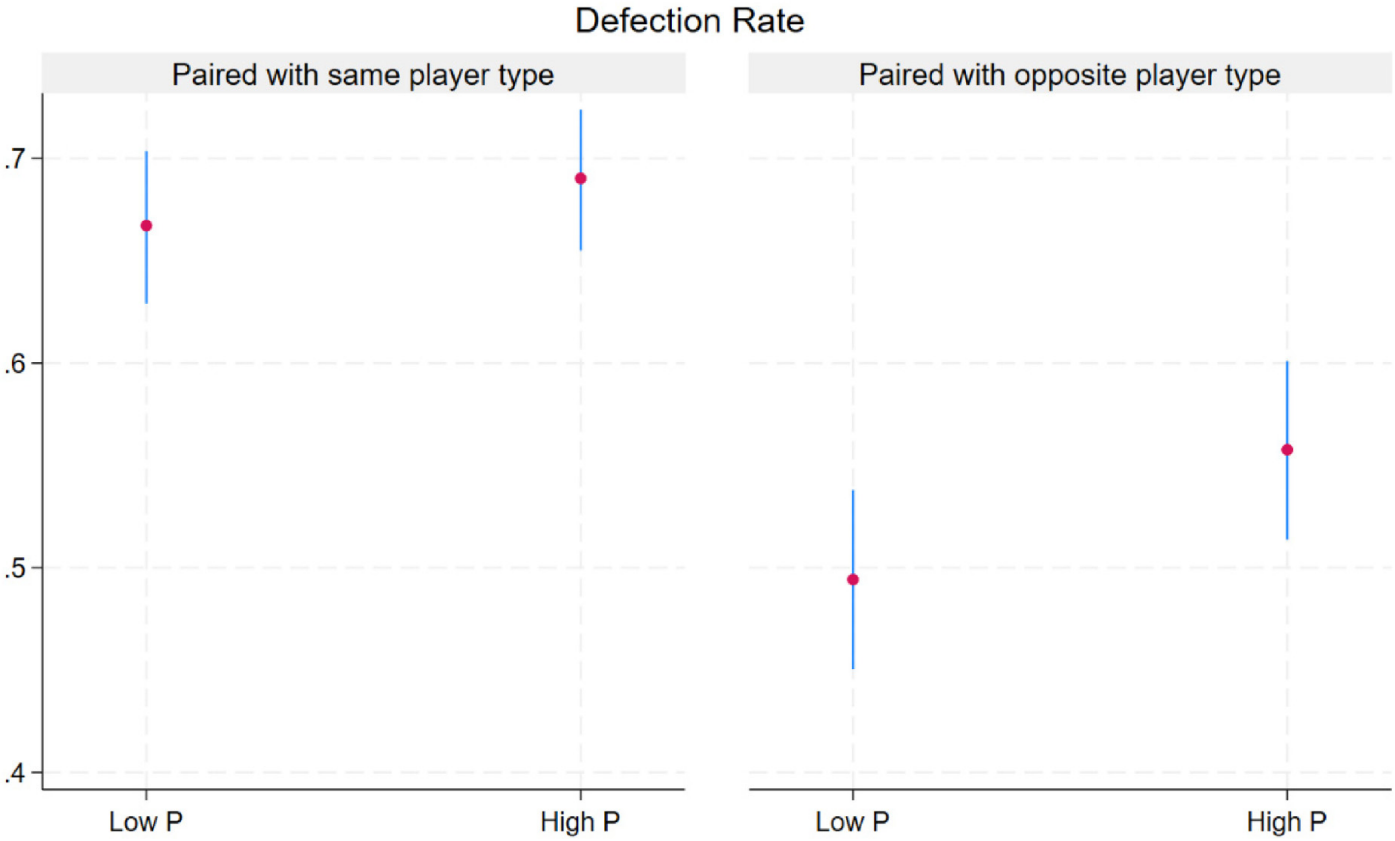
Mean defection rate (95% CI) over 2,400 rounds by match type and level of psychopathy.

**Figure 2.**
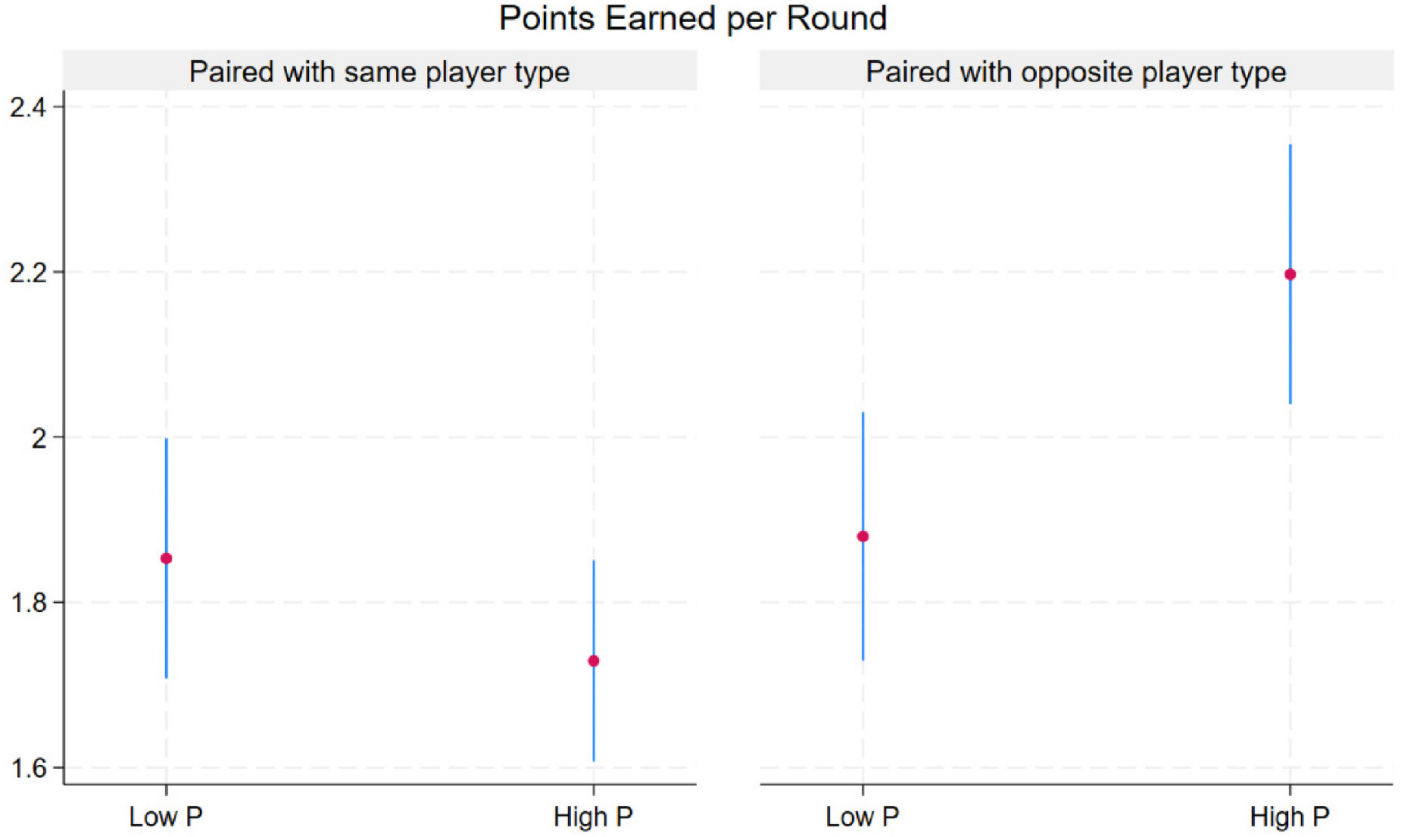
Mean points earned (95% CI) over 2,400 rounds by match type and level of psychopathy.

Descriptive statistics of defection rates and earnings by match and player type are presented visually in Figures 1 and 2. As shown in Table 3, opponent defection in a previous round increased the odds of defection (OR: 2.34, 95% CI: 1.50–3.67). Contrary to the first hypothesis, High PP traits (self or opponent) were not associated with defection odds. Mixed matches (High PP vs. Low PP or vice-versa) had lower odds of defection (OR: 0.47, 95% 0.25–0.90). Note the contrast of lower defection rates in the right panel versus the left panel of Figure 1.

**Table 3.** Logistic Regression Model of Defection.

Predictor	OR	Robust SE	z	*p*	95% CI
High PP (self)					
No	(Reference category)				
Yes	1.35	0.41	0.97	.33	0.74–2.47
High PP (opponent)					
No	(Reference category)				
Yes	0.87	0.27	−0.46	.65	0.48–1.59
Opponent defected in the previous round					
No	(Reference category)				
Yes	2.34	0.54	3.72	<.001	1.50–3.67
Mixed match					
No	(Reference category)				
Yes	0.47	0.16	−2.29	.02	0.25–0.90
Round (range: 1–40)	1.02	0.01	2.86	<.001	1.01–1.03
(Constant)	0.90	0.26	−0.36	.72	0.52–1.58
Variance components	Estimate	Robust SE			95% CI
SD of within person error	1.03	0.17			0.75–1.43
Intraclass correlation	0.25	0.06			0.14–0.38

After adjustment for opponent type, the player's previous move, the opponent's previous move, pair type, and round number, High PP players earned about a half-point more per round (0.54) compared to Low PP. However, High PP players lost more than one point (−1.04) per round when paired with another High PP player (Table 4). The same model showed that defection earned almost 2 points more (1.73) per round than cooperation.

**Table 4.** Linear Regression Model of Points Earned per Round.

Predictor	B	Robust SE	z	*p*	95% CI
High PP (self)					
No	(Reference category)				
Yes	0.54	0.26	2.12	.03	0.04–1.05
High PP (opponent)					
No	(Reference category)				
Yes	0.33	0.28	1.17	.24	−0.23–0.89
Opponent defected in the previous round					
No	(Reference category)				
Yes	0.06	0.16	0.36	.72	−0.26–0.38
Defected (self)					
No	(Reference category)				
Yes	1.73	0.14	12.07	<.001	1.45–2.01
Pair					
At least one player is Low P	(Reference category)				
Both High P	−1.04	0.40	−2.6	.01	−1.83–−0.25
Round (range: 1–40)	−0.01	0.00	−3.25	<.01	−0.02–−0.01
(Intercept)	0.96	0.20	4.81	<.001	0.57–1.35
Variance components	Estimate	Robust SE			95% CI
SD of within person error	0.65	0.08			0.52–0.82
SD of residuals	1.53	0.04			1.46–1.61
Intraclass correlation	0.15	0.03			0.1–0.23

## Computer Matches

The computer matches replicated the mixed or homogenous pairing in the human matches except that TFT or a variant thereof was added as a third player. This resulted in six possible matchups: (High PP vs. High PP, High PP vs. Low PP, High PP vs. TFT, Low PP vs. Low PP, Low-PP vs. TFT, and TFT vs. TFT). There were two reasons for adding TFT (or its variant) into the matches: (1) it was highly successful in Axelrod's computer tournaments and served as a benchmark; (2) having a third player allows for a larger field, potentially revealing rewarding and unrewarding matchups.

From the human matches, retaliatory and unprovoked defection probabilities for High PP and Low PP players were calculated. Retaliatory defection is defined as the probability of a player defecting when the opponent defected in the previous round. Unprovoked defection is the probability of defecting in the first round, and for subsequent rounds, the probability of defecting if the opponent previously cooperated (the bold entry in Round 6 of Table 5). These probabilities were calculated based on empirical probabilities of Low PP and High PP human players over 40 rounds in 15 matches (Table 6).

**Table 5.** Examples of Unprovoked and Retaliatory Defections.

	Round number							
	1	2	3	4	5	6	7	…
Player A	C	C	C	C	C	**D**	D	
Payer B	C	C	C	C	C	C	D	

C = cooperate; D = defect.

**Table 6.** Defection Probabilities by Strategy.

	Low PP	High PP	TFT^a^	Prodigal TFT^a^
Retaliatory	0.68	0.78	1	0.85
Unprovoked	0.45	0.42	0	0.15

a Note: Low PP and High PP played either TFT or Prodigal TFT in the matchups but not both simultaneously. Low PP and High PP did not differ significantly in unprovoked defection proportions (chi-squared = 2.1716, df = 1, *p* = .1406, but differed in retaliatory proportions (chi-squared = 30.323, df = 1, *p* < .001).

In Table 5, Player A defected in Round 6 even though Player B cooperated in rounds 1 to 5. Hence, Player A's defection in Round 6 counts as an unprovoked defection. Player B's defection in Round 7 is a retaliatory defection because Player A defected in Round 6. Because TFT always cooperates on the first move and always defects in response to an opponent's defection, its unprovoked defection probability is 0 and its retaliatory defection is 1 (Table 6). Inasmuch as TFT never succumbs to Temptation (the lower left corner of Table 1) and always retaliates after a defection, it does not mirror human reciprocity since people are more forgiving of opponent defection (e.g., Generous TFT or tit-for-two-tats) (Mokros et al., 2008; Wedekind & Milinski, 1996) and less cooperative in one-shot PDs (Rand & Nowak, 2013). Accordingly, we introduced a variant called Prodigal TFT, since it strays from TFT's “straight and narrow path.”

The virtual strategies were entered into round robin matches. In the first play, each strategy cooperates or defects based on each one's unprovoked defection probabilities (Table 6). Conditional on the opponent's first move, the virtual strategies made their next move. As shown in Table 7 we set the initial population frequencies as: Low PP (90%), TFT (or Prodigal TFT) (5%), and High PP (5%), of which the latter was based on an estimated prevalence of clinically diagnosed psychopathy (Sanz-Garcia et al., 2021). As noted in the Introduction, a dimensional interpretation of psychopathy would mean players selecting the selfish action a certain fraction of the time.

**Table 7.** Summary of the Virtual Strategy Matchups (Each With 50 Replicates).

	Condition 1: TFT as the Third Player	Condition 2: Prodigal TFT as the Third Player
Initial population shares (%)	Low PP (90%)	Low PP (95%)
	High PP (5%)	High PP (5%)
	TFT (5%)	Prodigal TFT (5%)
Winner	TFT	High PP
Mean number of generations to extinction (SD)	11,209 (363)	57,195 (3163)

The initial population frequencies increased (decreased) in proportion to how much a strategy exceeds (falls below) the average payoff in a generation, as described in the replicator dynamic equations (Nowak, 2006):

$$x_{n+1}=x_{n}\cdot s\cdot (a_{n}-\varphi_{n}\;)$$

$$y_{n+1}=y_{n}\cdot s\cdot (b_{n}-\;\varphi_{n})$$

In the above, *x* and *y* are any two players, $\varphi_{n}$ is the average payoff of all pairwise matches in one generation, *a* and *b* are the payoffs of Player *x* and Player *y*, and *n* is a generation. Each match consists of a random number of rounds between 1 and 40. The variable *s* is a scaling constant set to 1/100 that controls the rate of evolution. The matches continue until two of the three players become extinct, which was defined as two players falling below a combined 2% population share. To prevent excessively long simulations, the number of generations was limited to 200,000.

We repeated each condition of the computer matchups 50 times, starting with a different random seed. These seeds handle the stochastic nature of the unprovoked and retaliatory defection probabilities of the human-derived strategies (Table 6) and ensure that the results are reproducible. The R code (with contributions from Dr Stephen Benning) can be found at https://github.com/lloydxeno/PrisonerDilemma/blob/main/condition_1 and at https://github.com/lloydxeno/PrisonerDilemma/blob/main/condition_2. The human experimental data are available at https://zenodo.org/records/12667513.

## Results of the Computer Matches

In condition 1 of the computer matches, TFT drove both Low PP and High PP to extinction 100% of the time (Appendix III). In condition 2 of the computer matches, High PP drove both Low PP and Prodigal TFT 100% of the time (Appendix IV). The end results of the matches can be understood by examining the average payoff per generation (Table 8). The evolution of population shares over generations is visualized in Appendix V for Condition 1 and in Appendix VI for Condition 2.

**Table 8.** Expected Payoffs to the Row Player When Matched With the Column Player.^a^

Condition 1: TFT as Third Player			
	Low PP	High PP	TFT
Low PP	**80**	**74**	82
High PP	**81**	**75**	78
TFT	78	74	100

Condition 2: Prodigal TFT as Third Player			
	Low PP	High PP	Prodigal TFT
Low PP	80	74	85
High PP	82	75	83
Prodigal TFT	78	74	*86*

a These payoffs are over 40 rounds in a single generation.

## Discussion

We held human and computer PD matches to test the adaptive hypothesis of psychopathy. In the human matches, we found that Low PP students were as likely to defect as High PP counterparts, contrary to our hypothesis. We also found that High PP had higher earnings in human matches relative to Low PP but not when playing another High PP player. Defection (choosing the selfish action) was the strongest predictor of earnings in the regression model. In the computer matches, TFT dominated the other two strategies in all matchups, but High PP dominated the matches with Low PP and Prodigal TFT. The implications of our results are now discussed.

### Human Matches

Since psychopathy is on one end of the selfish-selfless continuum (Sonne & Gash, 2018) and altruism on the other we were surprised that the odds of defection did not differ between high and Low PP players. Notably, matches consisting of two Low PP players had the same overall defection odds as homogeneous matches with two High PP players (Figure 1 left panel). What distinguishes Low PP from High PP is the latter's higher propensity to retaliate to an opponent's defection. Unprovoked defection levels were similar. Our finding concurs with previous results that high PP individuals flexibly deploy cooperation and defection depending on situational factors such as opponent harshness (Baggio & Benning, 2022) or the promise of long-term rewards (Gervais et al., 2013).

What we found puzzling is the high defection odds in matches composed of two Low PP human players—defection was not significantly lower than in matches with High PP's playing each other. This could have been driven by a desire *not to lose* or perhaps *a lack of trust* in the opponent. Perhaps this was a defensive strategy: defecting guarantees a minimum of 1 point while cooperating risks a zero earning. From an evolutionary perspective, cooperation in the PD decreases one's fitness and increases the opponent's fitness (Roca et al., 2009). Still, it is surprising that Low PP human players had a higher ratio of unprovoked to retaliatory defections compared to High PP (Table 6).

That the highest defection odds were observed in the High PP-High PP matches is unsurprising given previously reported findings (Balafoutas et al., 2021; Johnston et al., 2014). This may have resulted from a combination of insensitivity to punishment and a desire to dominate the other party. Low fear makes highly PP individuals less sensitive to others’ pain (Blair, 2007) and to punishment (van Honk et al., 2003) although Baggio and Benning (2022) found that PP players respond to harsh punishment by opponents. Accordingly, PP individuals may prefer to bully weak individuals to gain social status with a low risk of retaliation (van Geel et al., 2017).

High PP's higher retaliatory defection rate can be viewed from the lens of reciprocal punishment. In its usual sense, reciprocity is taken in its positive form: repaying kindness with kindness (Guala, 2012), but repaying harm with harm is reciprocity just the same. This form of reciprocity comes without gain, so Guala calls it negative reciprocity. Incurring a cost without the prospect of a reward is why it is called altruistic punishment. Whether altruistic or not, punishments enforce cooperation in groups and without it, cooperation breaks down (Fehr & Gächter, 2002). Fraternities and organized crime rely on bullying and harsh punishment as a means of testing loyalty and enforcing discipline among its members (Cimino, 2012; Varese, 2011). Fehr and Gachter (2002) theorized that negative emotions towards defectors are one mechanism underlying altruistic punishment. We propose that aggressiveness in psychopathy promotes a desire to punish and low fear discounts the potential harm of retaliatory actions. This greater willingness to punish can dissuade defection, so paradoxically, psychopathy may promote cooperation and perhaps PP individuals become more cooperative themselves.

Revenge is probably what drives the greater propensity for punishment of High PP individuals. Psychopathy is closely related to both Machiavellianism and narcissism and these three constitute the Dark Triad (Paulhus & Williams, 2002). Hancock and colleagues (2023) studied these Dark Triad traits in the context of unmet expectations in an imagined service encounter scenario. Some encounters were experimentally manipulated to have a failure outcome and others a successful one. The dependent variable was one of three possible reactions to the failed outcome: avoiding the provider, vindictive complaining, and negative word of mouth. Psychopathy was unique in being directly related to a desire for revenge as a mediator, leading to vindictive complaining and negative word of mouth as reactions. In contrast to both Machiavellianism and narcissism, psychopathy was unrelated to avoidance. In another study, Rasmussen and Boon examined the Dark Triad in the context of imagined infidelity by a romantic partner. Psychopathy was negatively related to the belief that revenge would be costly and positively related to the belief that one would be effective at exacting revenge. The belief in one's capacity to carry it out increased the chance of taking revenge (Rasmussen & Boon, 2014). Taken together with our PD result, psychopathy probably serves as a deterrent to defection because defectors can expect to be punished severely.

PP traits tend to be expressed in conflict but can also be channeled toward prosocial actions such as helping a stranger or giving blood (Smith et al., 2013). These can be useful in risky occupations such as police work (Falkenbach et al., 2017). Psychopathy can also be economically advantageous. A labor market study found that people scoring higher in psychopathy were more likely to be employed in real estate, advertising, and management consulting (Lindley, 2018) compared to other occupations. These occupations require the ability to sell ideas or products which in turn depends on savvy interpersonal skills. In summary, PP traits without antisocial actions can be compatible with civil society.

### Computer Matches

Despite starting at a 5% initial frequency in all its matches, TFT drove both High PP and Low PP to extinction in all Condition 1 replications. To understand the Condition 1 result, note that TFT versus TFT matches are extremely rewarding for TFT. TFT's payoff from these matches (average payoff = 100) more than offsets its deficits in matches with Low PP and High PP. By comparison, High PP has the highest payoff when playing against itself (average payoff = 75) and against Low PP (average payoff = 81), but its margin over the other strategies is small. High PP has a deficit of 4 points to Low PP and 18 points to TFT when the opponent is TFT. The replicator dynamics equation compounds TFT's gain in each generation, with the result that it dominates the population.

The expected payoffs in a hypothetical two-player match between Low PP and High PP are shown in the 2 × 2 submatrix of Table 8 (cells with the bold font). This submatrix shows that High PP strictly dominates Low PP in a two-player match. However, if TFT joins the match as a third player, Low PP is liberated from strict dominance by High PP because Low PP earns more from matches with TFT (payoff = 82) than High PP earns from matches with TFT (payoff = 78). In effect, the presence of TFT allows Low PP to absorb its relative disadvantage against High PP. Unfortunately, Low PP also harms its long-term survival because the replicator dynamics equation guarantees TFT's dominance in the long run.

**Figure 3.**
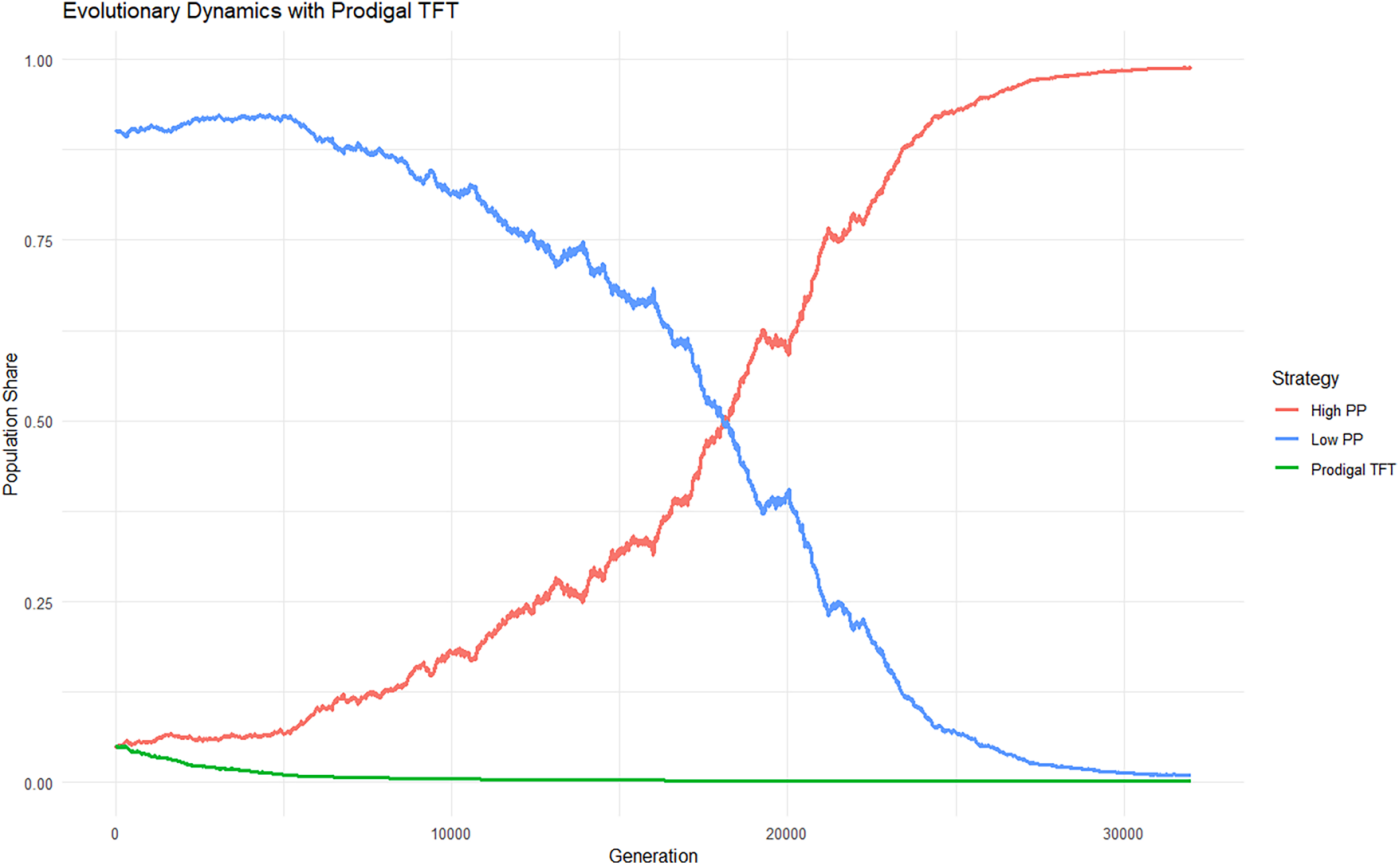
Evolution in population shares in a three-player Prisoner Dilemma.

In Condition 2, High PP drove Prodigal TFT and Low PP to extinction in all replications. The contrast of Prodigal TFT's failure and TFT's success against the same opponents is also revealed in Table 8. In Condition 2, Prodigal TFT playing a copy of itself has the highest expected payoff per generation (payoff = 86). Unfortunately, Prodigal TFT's surpluses in Condition 2 in matches against itself (+3 vs High PP and +1 vs Low PP) turn into a net deficit of 3 when combined with the result of matches against High PP (-1 vs High PP and 0 vs Low PP) and in matches against Low PP (-4 vs High PP and -2 vs Low PP), Observe that the numbers in the cells in Condition 2 are very similar to those in Condition 1, except that Prodigal TFT's expected payoff when playing a copy of itself is markedly smaller (payoff = 86 in italic font). This indicates that Prodigal TFT was both the agent and victim of its inferior performance relative to TFT. High PP's expected payoff across all matches is higher than that of Low PP, so High PP drives the other two strategies to extinction (Figure 3).

Why might a strategy like Prodigal TFT arise? The literature suggests that people generally follow a reciprocal strategy but deviate from it for one reason or another (Rand & Nowak, 2013; Wedekind & Milinski, 1996). When the Temptation payoff is increased, people who usually reciprocate tend to cooperate less (Blonski et al., 2011). Believing that the opponent cannot tell if one's defection was intentional or accidental can also motivate higher unprovoked defection (Falk et al., 2008). These and other reasons can give rise to Prodigal TFT. Condition 2 reveals the irony that the difference between TFT's dominance or extinction is a 15% change in unprovoked defection combined with a 15% change in retaliation probabilities. Cooperative individuals may sometimes try to optimize the balance of cooperation and defection for selfish reasons but end up with an inferior outcome in the long run. Cooperators who deviate from their usual practice present an opportunity for High PP to thrive and dominate the population.

### Synthesis of Human and Computer Matches

The complementarity of human and computer matches allowed each to address the limitation of the other. Human matches paired High PP with fellow High PP players and this led to the finding that High PP tends to earn less than it did in mixed pairs. Similarly, the computer matches showed that High PP had higher earnings over Low PP unless TFT joined as a third player. The computer matches further showed that starting from a low frequency in the population, TFT outcompetes both High PP and Low PP unless it becomes more selfish and less retaliatory. When this happens, High PP becomes the dominant strategy in the population. Taken together, these findings may explain why psychopathy persists. It cannot take over the population because when highly PP people interact, they tend to do worse compared to cooperative player types like TFT. TFT players may get burned by defectors and decide to adopt a more selfish strategy. In doing so, their victim could be High PP (which is self-defeating for both) or another TFT player—potentially triggering a cascade of defections. Fowler and Christakis (Fowler & Christakis, 2010) have shown that defection is socially contagious—reciprocal cooperators can reduce cooperation when they experience defection. This can be a purely defensive response: defection prevents further exploitation and is a key ingredient of TFT's success.

To prevent a collapse of cooperation, individuals probably have signals to distinguish between friend and foe. Trivers (1971) proposed that cooperators require such signals to avoid being parasitized by free-riders. These mechanisms include reputation, past behavior, and emotions. Psychopaths, on the other hand, may adopt opportunistic, single-shot interactions in a population of cooperators (Dugatkin & Wilson, 1991; Harpending & Sobus, 1987). Suppose there were only two behavioral strategies in a population: pure cooperators (always Cooperate) and pure defectors (always Defect). Here, there is a divergence of interests: while the former would prefer to meet their kind only in repeated encounters, selfish ones would prefer to meet cooperators only, preferably in one-shot encounters, to avoid retaliation. Signaling one's type, whether honestly or deceptively is fallible, raising the possibility of a disadvantageous match (Gintis et al., 2001). Failing to meet the “right” opponent is costly or suboptimal (Barclay, 2013). This situation can give rise to strategies that adopt a mixture of cooperation and defection. This leads us to suggest an alternative reading of Colman and Wilson's idea of a mixed strategy regarding psychopathy:

There are two possible interpretations of the mixed-strategy equilibrium in an evolutionary game of this type. Either the population evolves to a dimorphic mixture of cooperative and antisocial phenotypes … or it evolves to a form in which every individual shows a propensity to randomize between behaving cooperatively and antisocially in the required proportions. (Colman & Wilson, 1997, p. 31).

Colman and Wilson (1997) favored the first possibility, arguing that most people are not antisocial. But PP traits can also be transmitted by learning, and not just genetically. In a laboratory experiment, people who focused on the success of others tended to be more selfish compared to those who focused only on the others’ playing style without information about payoffs (van den Berg et al., 2015). The high odds of defection in Low PP versus Low PP human matches shows that everyone can act selfishly. Some instances of defection could be a form of trial-and-error, as exemplified in the Rock-Paper-Scissors game. In short, psychopathy may persist because everyone adjusts their level of selfishness or cooperation to the context, in pursuit of economic advantage.

Our study had several limitations. The participants were college students, so our findings do not generalize to clinically diagnosed psychopaths. Our sample size of 60 was relatively small compared with other studies of psychopathy, but this sample was obtained by a two-stage stratified design. This design allowed us maximal power by comparing the extreme quartiles of 448 university students. The groups did not differ in a proxy for intelligence, although the High PP group was about 2 years younger. The players were not interviewed after the PD to examine their motives for defecting, their emotions toward the other player, and other factors leading to their playing style. It would have been valuable to interrogate High PP players whether a retaliatory defection was a defensive strategy (to prevent being a sucker) or to punish the opponent (for a perceived slight). We focused on selfishness and willingness to punish, but we realize that psychopathy includes other traits such as superficial charm, shallow affect, lack of remorse, and pathological lying, which are not captured in the PD. Therefore, our findings should be taken as shining a light on two facets of psychopathy.

In summary, PP traits may proliferate in two ways. People with low psychopathy engage in selfish behaviors also and people with high psychopathy are capable of cooperation and deter defection by means of harsh punishment. The broader significance of the in silico experiment is that randomness in social encounters may allow behavioral expressions of psychopathy to thrive over an altruistic and reciprocal strategy such as TFT.

## Supplemental Material

sj-docx-1-evp-10.1177_14747049261435215 - Supplemental material for Propensity to Punish in High Psychopathy may Promote Cooperation: Human and Computer Prisoner Dilemma Experiments Supplemental material, sj-docx-1-evp-10.1177_14747049261435215 for Propensity to Punish in High Psychopathy may Promote Cooperation: Human and Computer Prisoner Dilemma Experiments by Lloyd Balbuena, Nathan Kolla and John Logan in Evolutionary Psychology

sj-docx-2-evp-10.1177_14747049261435215 - Supplemental material for Propensity to Punish in High Psychopathy may Promote Cooperation: Human and Computer Prisoner Dilemma Experiments Supplemental material, sj-docx-2-evp-10.1177_14747049261435215 for Propensity to Punish in High Psychopathy may Promote Cooperation: Human and Computer Prisoner Dilemma Experiments by Lloyd Balbuena, Nathan Kolla and John Logan in Evolutionary Psychology

sj-docx-3-evp-10.1177_14747049261435215 - Supplemental material for Propensity to Punish in High Psychopathy may Promote Cooperation: Human and Computer Prisoner Dilemma Experiments Supplemental material, sj-docx-3-evp-10.1177_14747049261435215 for Propensity to Punish in High Psychopathy may Promote Cooperation: Human and Computer Prisoner Dilemma Experiments by Lloyd Balbuena, Nathan Kolla and John Logan in Evolutionary Psychology

sj-docx-4-evp-10.1177_14747049261435215 - Supplemental material for Propensity to Punish in High Psychopathy may Promote Cooperation: Human and Computer Prisoner Dilemma Experiments Supplemental material, sj-docx-4-evp-10.1177_14747049261435215 for Propensity to Punish in High Psychopathy may Promote Cooperation: Human and Computer Prisoner Dilemma Experiments by Lloyd Balbuena, Nathan Kolla and John Logan in Evolutionary Psychology

sj-docx-5-evp-10.1177_14747049261435215 - Supplemental material for Propensity to Punish in High Psychopathy may Promote Cooperation: Human and Computer Prisoner Dilemma Experiments Supplemental material, sj-docx-5-evp-10.1177_14747049261435215 for Propensity to Punish in High Psychopathy may Promote Cooperation: Human and Computer Prisoner Dilemma Experiments by Lloyd Balbuena, Nathan Kolla and John Logan in Evolutionary Psychology

sj-docx-6-evp-10.1177_14747049261435215 - Supplemental material for Propensity to Punish in High Psychopathy may Promote Cooperation: Human and Computer Prisoner Dilemma Experiments Supplemental material, sj-docx-6-evp-10.1177_14747049261435215 for Propensity to Punish in High Psychopathy may Promote Cooperation: Human and Computer Prisoner Dilemma Experiments by Lloyd Balbuena, Nathan Kolla and John Logan in Evolutionary Psychology
