# Supplementary material for: Propensity to Punish in High Psychopathy may Promote Cooperation: Human and Computer Prisoner Dilemma Experiments
Source: Evol Psychol. 2026 Mar 21;24(1):14747049261435215. doi: 10.1177/14747049261435215 (PMC13009891; doi:10.1177/14747049261435215)
Supplement: sj-docx-1-evp-10.1177_14747049261435215 - Supplemental material for Propensity to Punish in High Psychopathy may Promote Cooperation: Human and Computer Prisoner Dilemma Experiments [file sj-docx-1-evp-10.1177_14747049261435215.docx]

**Appendix I: Instructions for the Tower of Hanoi**


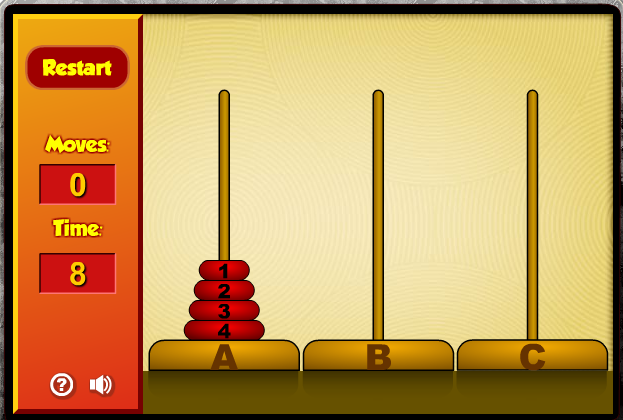


The objective of this game is to move the rings on the left pole to the right pole in as few moves as possible. You can move any ring to any pole subject to the following constraints:

1. You can move only one ring at a time—the uppermost one in a stack.
2. You cannot put a bigger ring on top of a smaller one.

You move a ring by clicking on it and clicking on the pole of your choice. Try the task with 3 rings for practice to get a feel of the game. This practice round will not be recorded. Do you have any questions?

{Pause and let participants solve the 3-ring problem}

Now that you are familiar with the task, the first round will be a 4-ring problem. The rules are the same. Start whenever you are ready. Let me know when you finish so I can record the number of moves it took for you.

{After finishing the round}

The second round will be a 5-ring problem The rules are the same. Start whenever you are ready. Let me know when you finish so I can record the number of moves it took for you.
