# Supplementary material for: Propensity to Punish in High Psychopathy may Promote Cooperation: Human and Computer Prisoner Dilemma Experiments
Source: Evol Psychol. 2026 Mar 21;24(1):14747049261435215. doi: 10.1177/14747049261435215 (PMC13009891; doi:10.1177/14747049261435215)
Supplement: sj-docx-2-evp-10.1177_14747049261435215 - Supplemental material for Propensity to Punish in High Psychopathy may Promote Cooperation: Human and Computer Prisoner Dilemma Experiments [file sj-docx-2-evp-10.1177_14747049261435215.docx]

Appendix II: **Instructions for the Prisoner Dilemma Game**

In Game #1 you will be playing a computer game against each other (the other student in the pair) for 40 rounds. The game is played on a computer terminal in which you are paired with another student and your task is to select a move “S” or “C”. Take a look at your monitor and find the message that tells you whether you are the row or column player.


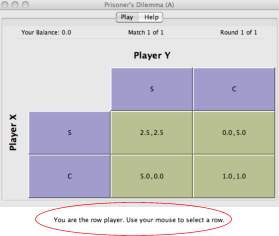


Figure 1: Before the player moves

Now hover your mouse over a column (or row) and notice that your payoff appears in bold font. Try moving your mouse to the other choice and notice that your payoff appears in bold as a result. Notice that your payoff differs for each move and also differs depending on the move of the other player, which you will not know until the end of a round.


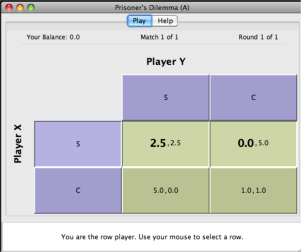


Figure 2: Row player hovers on "S" row and payoffs are highlighted.

To select a move, click on the a row (or column). Once you click on a choice, you cannot change it so make sure you choose well. After you and your partner have both moved, you will get feedback about the amount you won. Take note especially of the figure on the upper left hand corner of the window which shows your accumulated winnings so far. Your winnings at the end of the 40 rounds will be converted to its equivalent value in cents (1 point = 1 cent).


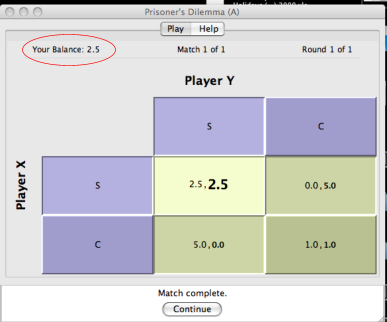


**Figure 3:** Screen showing accumulated amount won.

I will now test to your understanding of the logic of the game.

(1) Suppose the Row Player in [in Figure 3] plays “S” and the Column Player, plays “C”, how much does the Row Player win? {0}. The Column Player? {5}

(2) In a pair of numbers like {2.5, 0}, how much is the column player’s payoff? {0}

(3) Are you allowed to communicate your intended choice to the other? {No.}

We will now play 5 test-rounds to familiarize you with the game.
