# Supplementary material for: Propensity to Punish in High Psychopathy may Promote Cooperation: Human and Computer Prisoner Dilemma Experiments
Source: Evol Psychol. 2026 Mar 21;24(1):14747049261435215. doi: 10.1177/14747049261435215 (PMC13009891; doi:10.1177/14747049261435215)
Supplement: sj-docx-3-evp-10.1177_14747049261435215 - Supplemental material for Propensity to Punish in High Psychopathy may Promote Cooperation: Human and Computer Prisoner Dilemma Experiments [file sj-docx-3-evp-10.1177_14747049261435215.docx]

**Appendix III: Prisoner Dilemma Round-robin Match-up Results among TFT, High PP, and Low PP**

| Replicate | Generations to Extinction | High PP Final | TFT Final | Low PP Final |
| --- | --- | --- | --- | --- |
| 1 | 10658 | 0.005 | 0.985 | 0.010 |
| 2 | 11346 | 0.005 | 0.985 | 0.010 |
| 3 | 11698 | 0.006 | 0.984 | 0.010 |
| 4 | 11187 | 0.006 | 0.984 | 0.010 |
| 5 | 10847 | 0.006 | 0.984 | 0.010 |
| 6 | 11135 | 0.005 | 0.985 | 0.010 |
| 7 | 10917 | 0.005 | 0.985 | 0.010 |
| 8 | 11570 | 0.005 | 0.985 | 0.010 |
| 9 | 11043 | 0.006 | 0.984 | 0.010 |
| 10 | 10920 | 0.006 | 0.984 | 0.010 |
| 11 | 11624 | 0.006 | 0.984 | 0.010 |
| 12 | 10915 | 0.006 | 0.984 | 0.010 |
| 13 | 11154 | 0.006 | 0.984 | 0.010 |
| 14 | 10563 | 0.005 | 0.985 | 0.010 |
| 15 | 11531 | 0.006 | 0.984 | 0.010 |
| 16 | 11240 | 0.006 | 0.984 | 0.010 |
| 17 | 11554 | 0.005 | 0.985 | 0.010 |
| 18 | 11429 | 0.006 | 0.984 | 0.010 |
| 19 | 11422 | 0.005 | 0.985 | 0.010 |
| 20 | 10700 | 0.006 | 0.984 | 0.010 |
| 21 | 11203 | 0.005 | 0.985 | 0.010 |
| 22 | 11938 | 0.006 | 0.985 | 0.010 |
| 23 | 11071 | 0.006 | 0.984 | 0.010 |
| 24 | 11314 | 0.005 | 0.985 | 0.010 |
| 25 | 10798 | 0.005 | 0.985 | 0.010 |
| 26 | 10998 | 0.005 | 0.985 | 0.010 |
| 27 | 10951 | 0.006 | 0.984 | 0.010 |
| 28 | 10760 | 0.006 | 0.984 | 0.010 |
| 29 | 11697 | 0.005 | 0.985 | 0.010 |
| 30 | 11575 | 0.005 | 0.985 | 0.010 |
| 31 | 11159 | 0.005 | 0.985 | 0.010 |
| 32 | 11202 | 0.005 | 0.985 | 0.010 |
| 33 | 10545 | 0.005 | 0.985 | 0.010 |
| 34 | 11445 | 0.005 | 0.985 | 0.010 |
| 35 | 11010 | 0.006 | 0.984 | 0.010 |
| 36 | 11352 | 0.005 | 0.985 | 0.010 |
| 37 | 11459 | 0.006 | 0.984 | 0.010 |
| 38 | 11300 | 0.006 | 0.984 | 0.010 |
| 39 | 11924 | 0.005 | 0.985 | 0.010 |
| 40 | 11244 | 0.005 | 0.985 | 0.010 |
| 41 | 10979 | 0.006 | 0.984 | 0.010 |
| 42 | 11776 | 0.005 | 0.985 | 0.010 |
| 43 | 11546 | 0.006 | 0.984 | 0.010 |
| 44 | 11452 | 0.005 | 0.985 | 0.010 |
| 45 | 11385 | 0.006 | 0.984 | 0.010 |
| 46 | 11023 | 0.005 | 0.985 | 0.010 |
| 47 | 10295 | 0.005 | 0.985 | 0.010 |
| 48 | 10889 | 0.005 | 0.985 | 0.010 |
| 49 | 11225 | 0.005 | 0.985 | 0.010 |
| 50 | 11482 | 0.005 | 0.985 | 0.010 |
