# Supplementary material for: Propensity to Punish in High Psychopathy may Promote Cooperation: Human and Computer Prisoner Dilemma Experiments
Source: Evol Psychol. 2026 Mar 21;24(1):14747049261435215. doi: 10.1177/14747049261435215 (PMC13009891; doi:10.1177/14747049261435215)
Supplement: sj-docx-4-evp-10.1177_14747049261435215 - Supplemental material for Propensity to Punish in High Psychopathy may Promote Cooperation: Human and Computer Prisoner Dilemma Experiments [file sj-docx-4-evp-10.1177_14747049261435215.docx]

**Appendix IV: Prisoner Dilemma Round-robin Match-up Results among Prodigal TFT, High PP, and Low PP**

| Replicate | Generations to Extinction | High PP Final | Prodigal TFT Final | Low PP Final |
| --- | --- | --- | --- | --- |
| 1 | 32007 | 0.99 | < .01 | 0.01 |
| 2 | 27222 | 0.99 | < .01 | 0.01 |
| 3 | 24316 | 0.99 | < .01 | 0.01 |
| 4 | 28209 | 0.99 | < .01 | 0.01 |
| 5 | 31891 | 0.99 | < .01 | 0.01 |
| 6 | 35147 | 0.99 | < .01 | 0.01 |
| 7 | 30656 | 0.99 | < .01 | 0.01 |
| 8 | 33836 | 0.99 | < .01 | 0.01 |
| 9 | 27614 | 0.99 | < .01 | 0.01 |
| 10 | 35815 | 0.99 | < .01 | 0.01 |
| 11 | 26767 | 0.99 | < .01 | 0.01 |
| 12 | 30342 | 0.99 | < .01 | 0.01 |
| 13 | 31205 | 0.99 | < .01 | 0.01 |
| 14 | 33982 | 0.99 | < .01 | 0.01 |
| 15 | 32089 | 0.99 | < .01 | 0.01 |
| 16 | 31416 | 0.99 | < .01 | 0.01 |
| 17 | 33584 | 0.99 | < .01 | 0.01 |
| 18 | 29375 | 0.99 | < .01 | 0.01 |
| 19 | 34357 | 0.99 | < .01 | 0.01 |
| 20 | 32249 | 0.99 | < .01 | 0.01 |
| 21 | 34489 | 0.99 | < .01 | 0.01 |
| 22 | 27311 | 0.99 | < .01 | 0.01 |
| 23 | 35091 | 0.99 | < .01 | 0.01 |
| 24 | 31859 | 0.99 | < .01 | 0.01 |
| 25 | 32658 | 0.99 | < .01 | 0.01 |
| 26 | 32179 | 0.99 | < .01 | 0.01 |
| 27 | 26171 | 0.99 | < .01 | 0.01 |
| 28 | 27483 | 0.99 | < .01 | 0.01 |
| 29 | 31055 | 0.99 | < .01 | 0.01 |
| 30 | 36165 | 0.99 | < .01 | 0.01 |
| 31 | 32925 | 0.99 | < .01 | 0.01 |
| 32 | 32942 | 0.99 | < .01 | 0.01 |
| 33 | 31114 | 0.99 | < .01 | 0.01 |
| 34 | 31660 | 0.99 | < .01 | 0.01 |
| 35 | 32373 | 0.99 | < .01 | 0.01 |
| 36 | 30523 | 0.99 | < .01 | 0.01 |
| 37 | 31908 | 0.99 | < .01 | 0.01 |
| 38 | 30366 | 0.99 | < .01 | 0.01 |
| 39 | 31608 | 0.99 | < .01 | 0.01 |
| 40 | 34609 | 0.99 | < .01 | 0.01 |
| 41 | 31670 | 0.99 | < .01 | 0.01 |
| 42 | 28581 | 0.99 | < .01 | 0.01 |
| 43 | 34135 | 0.99 | < .01 | 0.01 |
| 44 | 31811 | 0.99 | < .01 | 0.01 |
| 45 | 33330 | 0.99 | < .01 | 0.01 |
| 46 | 34356 | 0.99 | < .01 | 0.01 |
| 47 | 33348 | 0.99 | < .01 | 0.01 |
| 48 | 33746 | 0.99 | < .01 | 0.01 |
| 49 | 32517 | 0.99 | < .01 | 0.01 |
| 50 | 29740 | 0.99 | < .01 | 0.01 |
