# Supplementary figures and images for: Propensity to Punish in High Psychopathy may Promote Cooperation: Human and Computer Prisoner Dilemma Experiments
Source: Evol Psychol. 2026 Mar 21;24(1):14747049261435215. doi: 10.1177/14747049261435215 (PMC13009891; doi:10.1177/14747049261435215)

**Appendix V: Evolution of Population Shares By Generation in Condition 1**

**
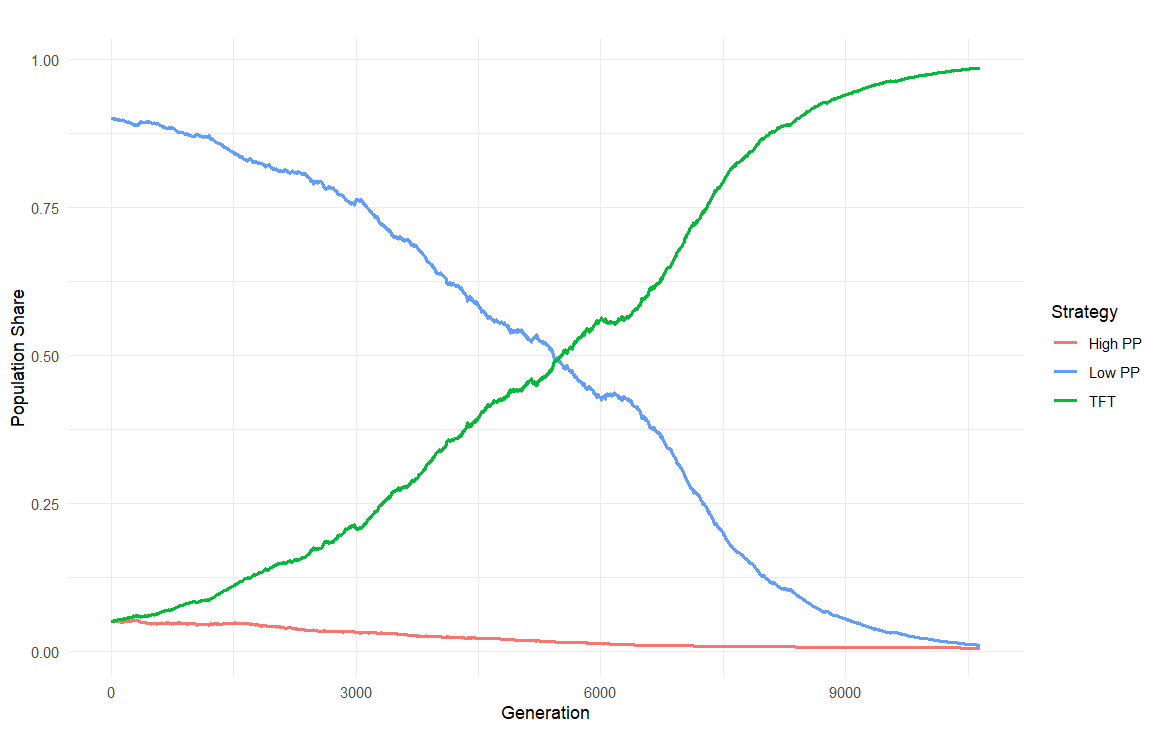
**

Supplement: sj-docx-5-evp-10.1177_14747049261435215 - Supplemental material for Propensity to Punish in High Psychopathy may Promote Cooperation: Human and Computer Prisoner Dilemma Experiments [file sj-docx-5-evp-10.1177_14747049261435215.docx]
